# Field Explosives Detectors—Current Status and Development Prospects

**DOI:** 10.3390/s25196024

**Published:** 2025-10-01

**Authors:** Dariusz Augustyniak, Mateusz Szala

**Affiliations:** Faculty of New Technologies and Chemistry, Department of Explosives, Military University of Technology, S. Kaliskiego 2, 00-908 Warsaw, Poland; mateusz.szala@wat.edu.pl

**Keywords:** explosives, detection, detectors, analysis, field detectors

## Abstract

**Highlights:**

**What are the main findings?**

- A review of ~80 commercially available mobile explosive detectors revealed wide technological diversity, from IMS and FTIR to GC–MS and QCM.
- Only a few devices use two orthogonal techniques, significantly improving detection reliability and reducing false alarms.

**What is the implication of the main finding?**

- Multi-technique approaches are essential to enhance explosive detection’s accuracy, efficiency, and robustness in real-world scenarios.
- The results provide practical guidance for selecting and developing portable detection systems for security, defense, and emergency response.

**Abstract:**

This review critically evaluates the performance of approximately 80 commercially available mobile detectors for explosive identification. The majority of devices utilize Ion Mobility Spectrometry (IMS), Fourier Transform Infrared Spectroscopy (FTIR), or Raman Spectroscopy (RS). IMS-based instruments, such as the M-ION (Inward Detection), typically achieve sensitivities at the ppt level, while other IMS implementations demonstrate detection ranges from low ppb to ppm. Gas Chromatography–Mass Spectrometry (GC–MS) systems, represented by the Griffin™ G510 (Teledyne FLIR Detection), provide detection limits in the ppb range. Transportable Mass Spectrometers (Bay Spec) operate at ppb to ppt levels, whereas Laser-Induced Fluorescence (LIF) devices, such as the Fido X4 (Teledyne FLIR Detection), achieve detection at the nanogram level. Quartz Crystal Microbalance (QCM) sensors, exemplified by the EXPLOSCAN (MS Technologies Inc. 8609 Westwood Center Drive Suite 110, Tysons Corner, VA, USA), typically reach the ppb range. Only four devices employ two orthogonal analytical techniques, enhancing detection reliability and reducing false alarms. Traditional colorimetric tests based on reagent–analyte reactions remain in use, demonstrating the continued relevance of simple yet effective methods. By analyzing the capabilities, limitations, and technological trends of current detection systems, this study underscores the importance of multi-technique approaches to improve accuracy, efficiency, and operational effectiveness in real-world applications. The findings provide guidance for the development and selection of mobile detection technologies for security, defense, and emergency response.

## 1. Introduction

The security of citizens of modern countries is one of the priorities of military and police services. A state of war as a state of emergency has its specificity, and the service’s actions are apparent. Security becomes an absolute priority, more important than economic development and general prosperity. In peacetime, the activities of the military, police, and secret services are not always obvious and, therefore, visible to the public. In this context, specialized tools such as portable hazardous materials detectors are an essential element affecting the efficiency of the service efforts. The frequent use of explosives as a means to carry out an act of diversion or terror is still observed. Preparing an improvised explosive device (IED) from commercially available materials is far simpler than using another hazardous agent, such as a biological agent.

In addition, the development of fireworks has led to the fact that explosive mixtures can be acquired without the applicable permits simply because they are adequately packaged and, if used correctly, should not cause a danger to health and life. The facilitated availability of fireworks carries the risk of using explosive mixtures from fireworks to construct an improvised explosive device (IED) by terrorists.

The processes described above have made it necessary for security services to use increasingly sophisticated hazardous materials detectors to quickly decide whether they are dealing with an attack or an accidental release of a legally marketed substance.

The monographs that continue to appear show that interest in the problem of detecting trace amounts of explosives is not waning. Attention is drawn to the fact that the authors are increasingly focusing on the issue of miniaturization of devices based on well-known as well as niche analytical techniques [1,2].

Over the past decades, scientific communities have conducted intensive research to develop new types of explosive sensors with enhanced sensitivity, selectivity, and suitability for portable applications. These efforts aim to design detection materials and systems that can be reliably operated under field conditions, ensuring rapid responses, robustness, and low production costs, which will be readily implemented into explosives detectors. Selected representative directions for this research are presented below.

Recently, a shift has been observed toward developing biological detection systems. Among these, biosensors utilizing recombinant microorganisms have been regarded as a promising alternative due to their selectivity, low production costs, and potential for safe deployment. A notable example is provided by the construction of a portable optical biosensor in which *Escherichia coli* cells have been engineered to emit bioluminescence in response to 2,4-dinitrotoluene (2,4-DNT), a volatile impurity of trinitrotoluene (TNT) that is commonly found in landmine vapors. A photodiode has detected the emitted signal and has been converted into an electrical output, enabling quantification of the analyte concentration. Prototypes of these biosensors have been shown to detect gaseous 2,4-DNT at concentrations down to 50 ppb, and their potential as innovative tools for landmine detection and environmental monitoring has been demonstrated [3].

Recent advancements in optoelectronic sensors have been highlighted by functionalizing titanium dioxide–based nanostructures with specific dopants, enhancing sensitivity and selectivity toward different classes of explosives. A promising direction has been demonstrated by the integration of polyoxometalates into TiO_2_ nanocrystals, through which rapid and selective detection of triacetone triperoxide (TATP) under variable light excitation has been enabled, with high stability and low detection limits being achieved [4].

Complementarily, the optical absorption of TiO_2_ has been extended into the visible range by functionalization with 5-amino-1,10-phenanthroline (Aphen), through which the formation of Meisenheimer complexes with nitroaromatic explosives such as TNT, DNT, and picric acid has been facilitated. This modification has improved charge carrier separation, enabling fingerprint-like recognition patterns of explosive vapors within seconds [5].

Through these approaches, the possibility of constructing single-sensor arrays capable of distinguishing between improvised and military explosives has been illustrated by the tuning of the surface chemistry and electronic properties of TiO_2_-based nanomaterials. This approach has paved the way for the development of portable, low-cost, and contactless detection systems suitable for field deployment. These studies are expected to contribute to developing novel explosive detectors.

Explosive mixtures used in improvised explosive devices have complex chemical compositions, which makes their detection with portable devices very difficult. However, in recent years, portable detectors have been developed to enable the identification of materials in the field. A special class comprises detectors that meet the MIL-STD 810H standard of the US Department of Defense [6]. These sensors are characterized by increased robustness. The detectors are designed primarily for military use. Meeting the requirements of MIL STD 810H ensures that the detector can withstand harsh operating conditions such as rain, dust, salt spray, and is resistant to drops and vibration.

The increased number of more sophisticated mobile detectors based on various analytical techniques has attracted the attention of the scientific research community. This interest is reflected in the growing number of publications in specialized periodicals. Available commercial explosives detector solutions are being reviewed by research teams. The available studies provide a comprehensive analysis of the current state of knowledge and future developments, and a presentation of the difficulties and limitations of the technique used by the detector [7,8,9,10].

Review studies of a different kind, which can be regarded as a guide to the commercial market for detectors, are prepared by law enforcement officers. A comprehensive compilation of publications is contained in Interpol’s studies [11,12]. A brief overview of selected explosive detectors has been prepared by the National Urban Security Technology Laboratory (Manhattan, NY, USA, ), with instruments compared in terms of operational capabilities [13].

This paper is a review of commercially available portable detectors and color tests. The primary criterion for selection was the ability to detect explosives. The classification of the devices was conducted based on the techniques they use. The information presented in the tables was sourced from data provided by the manufacturers/distributors, even if it was presented in a non-scientific manner.

## 2. Ion Mobility Spectrometry (IMS)

The attempts to construct a so-called artificial nose have been ongoing for decades [14]. The term “artificial nose” is applied to a set of chemical detectors that respond to different types of molecules in the environment or other specific characteristics of these molecules.

The analytical technique with the most significant potential to mimic the sense of smell is Ion Mobility Spectrometry. Due to its high potential for miniaturization and ubiquitous water, which does not affect measurements as negatively as classical mass spectrometry techniques, the IMS technique is readily used in mobile detectors.

Ion Mobility Spectrometry (IMS) is an analytical technique that separates ionized molecules in the gas phase based on their mobility under the influence of an electric field. The mobility of ions depends on their mass, shape, and charge, which allows for identifying analytes by measuring their drift time through a carrier gas. The resulting spectra—typically in drift time or compensation voltage plots—serve as characteristic fingerprints for specific chemical compounds.

In recent years, research has focused on a specific variant of Ion Mobility Spectrometry (IMS), known as Differential Ion Mobility Spectrometry (DMS), which separates ions using alternating high and low electric fields based on nonlinear mobility differences.

Electronic nose (e-nose) technology developments have introduced sensor arrays with chemically functionalized surfaces. A 16-channel system based on micro-capacitors coated with silane layers has demonstrated the high ability to detect TNT, DNT, and RDX [15]. Machine learning algorithms, such as Random Forest, have been successfully applied to classify sensor response patterns and distinguish explosive compounds from other chemicals [16].

Detecting trace amounts of explosives, including improvised compounds such as Triacetone Triperoxide (TATP) and HexaMethylene Triperoxide Diamine (HMTD), remains a critical challenge in public safety applications. Research on DMS, including the identification of (TATP) and Hexamethylene Triperoxide Diamine (HMTD), was conducted by M. Maziejuk et al. [17]. DMS enables effective identification of these substances, and adding ammonia to the carrier gas has been shown to enhance selectivity by suppressing interference from acetone, a common by-product in TATP synthesis.

In stationary systems, such as walk-through portals, dual DMS detectors—one with a semi-permeable membrane and one without—allow for detecting both high- and low-vapor-pressure explosives. Air samples are collected from the tested individual’s hands, pockets, and shoes, with a total analysis time of less than 5 s and no need to physically enclose the person during screening [18].

An analysis of the market for mobile detectors using the IMS technique has identified some 15 devices, the most critical operational parameters of which are collected in Table 1.

**Figure 1:**
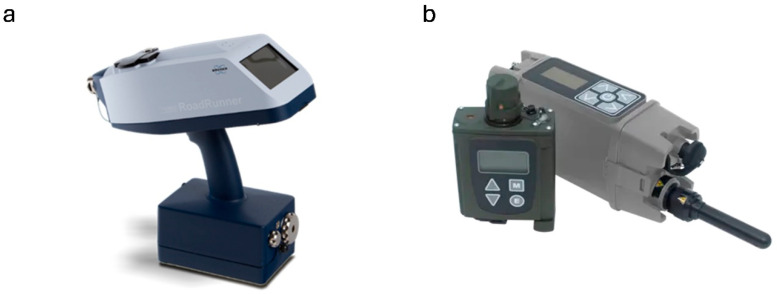
(**a**) RoadRunner explosives detector from Bruker [27]; (**b**) LCD 4 detector with XID adapter from Smith’s Detection [25].

The world’s leading analytical instrument manufacturers, such as Bruker and Smith’s Detection (USA), have a range of entire product lines. Still, many companies with smaller portfolios or even startups and spin-offs offer just one instrument model. Few companies provide detailed information about the detection range of their explosive detection devices. The device can display the detection result differently, i.e., from classes of chemical compounds to specific subclasses of chemical compounds, or even individual explosive names. In military and police applications, different devices are used to detect explosives. In the case of first responders or pyrotechnists, simply identifying the presence of an explosive is usually sufficient to initiate appropriate pyrotechnic procedures. Based on the number of compounds the device is declared to detect, Bruker’s RoadRunner performs the best in the comparison (Figure 1a).

The limits of detection of declared chemicals are stated by producers in very different ways, making comparison of devices in terms of this parameter difficult. Most devices detect nanograms of substance (ng), and one manufacturer declared picograms (pg) the limit. Some suppliers characterize the devices by expressing detection limits in the ppm, ppb, and ppt ranges. With the extraordinary capabilities of IMS technology and modern programmable electronics, the stated ranges can be considered realistic. The declared weight of the devices ranges from 0.73 kg to 10.8 kg. Such a wide range of device weights is due to several factors. The first is the capabilities of the detector. The more general the information the detector provides, the smaller its interface/display. The second factor is the peripheral accessories and the size of the battery. The third factor is the device’s resistance to environmental factors. Building a detector to military standards usually results in increased weight.

## 3. Raman Spectroscopy

Over the past few years, devices based on Raman spectroscopy have gained tremendous popularity. This is because the Raman spectrum recorded in the 200–4000 cm^−1^ range is a kind of “fingerprint” of the chemical compound, allowing rapid and reliable identification of explosives. It should be noted that portable/field spectrometers usually use the 200–3000 cm^−1^ range.

Raman spectroscopy is an attractive analytical technique due to the lack of need for sample preparation and the minimal procedures required before detection. Samples can be analyzed in their natural state, which translates into reduced analysis time. In addition, analysis with a Raman spectrometer allows non-invasive and non-destructive examination of samples, which is particularly important in the case of small amounts of samples that can then be submitted for laboratory testing. Another advantage is the ability to analyze samples in various states of matter: liquid, solid, solutions, suspensions, or pastes. The measurement is not limited by temperature or pressure range. An important aspect is the possibility of directly determining compounds in glass and plastic packages without opening them [37].

Throughout the last few years, there has been a rapid increase in commercially available handheld Raman spectrometers. Many detectors offer similar operational and design capabilities. Table 2 summarizes the data for 36 Raman spectrometers, which represent market trends according to the authors. In recent years, new measurement techniques such as Surface-Enhanced Raman Spectroscopy (SERS), Spatially Offset Raman Spectroscopy (SORS), low-frequency Raman spectroscopy (LF-Raman), and others have been worked on for implementation by manufacturers [38].

A review of the available Raman spectrometers on the market leads to the conclusion that the differences between the various devices are based on their size, the operating wavelength of the excitation laser, optical system solutions, measurement range, and resolution. Detection capabilities generally depend on the manufacturer’s assumptions of the end user and whether the offering is targeted at industry, public administrations responsible for citizen security, or the military sector. Many manufacturers do not declare a detection limit. Nevertheless, it has been noted that if such information exists, it is at the ng or mg levels, as in the case of detectors from Metrohm, Pendar Technologies, InPhotonics Inc., or Alakai Defense Systems. In general, manufacturers indicate performance parameters such as the range of electromagnetic radiation to be detected, the frequency of the laser, or the resolution of the recorded spectrum.

**Figure 2:**
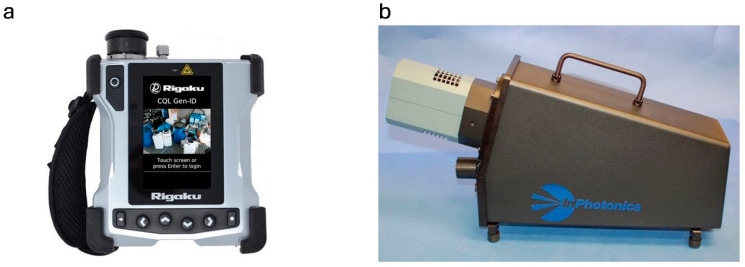
(**a**) CQL Gen-ID Raman spectrometer from Rigaku Analytical Devices [45]; (**b**) InPochelle Raman spectrometer from InPhotonics [68].

The physical characteristics of the devices, generally handheld Raman spectrometers weighing from a few hundred grams to several kilograms, as in the case of, for example, Bruker Corporation, Rigaku Analytical Devices (Figure 2a), Thermo Fisher, and Metrohm. Less common are portable spectrometers built in the form of a portable case. In this case, the weight can be as much as several kilograms for devices from companies Coda Devices Inc., InPhotonics Inc., or Optosky Photonics Inc. The last type is devices that can be mounted on mobile platforms typically used in the field; examples include the spectrometers from the following companies: Advanced Nano Technologies and Agilent Technologies. Based solely on the performance parameters declared by manufacturers, it is difficult to unequivocally indicate which of the detectors listed in Table 2 is best for field detection of explosives. The InPhochelle instrument from InPhotonics Inc. (Figure 2b) characterizes the broadest spectral range combined with the best resolution of recorded spectra.

### Surface-Enhanced Raman Scattering Technique

Surface-Enhanced Raman Spectroscopy (SERS) allows extension of the application of Raman Spectroscopy to trace analysis of explosives. The SERS technique increases detection sensitivity by improving the signal-to-noise ratio (S/N) by up to a million times. The described method is based on the optical properties of nanostructured metal-containing substrates. The most commonly used are gold and silver nanoparticles. Copper, platinum, and palladium are other possible applications for SERS. A sample of analyte is applied to suitably prepared surfaces. The interaction of the analyte with the substrate gives an enhanced signal to the Raman spectrometer. The analysis can be carried out with commercially available Raman spectrometers. Nevertheless, the result depends on the wavelength of the laser embedded in the spectrometer, the range of radiation, and the resolution [76]. Two research directions are observed in the literature on the SERS technique. One is testing of commercially available SERS substrates, and the other is the research work of scientific centers on new SERS substrates that enhance the analytical capabilities of this technique [77,78]. A review of sources for the current range of commercially available SERS substrates indicates that Thermo Fisher is intensively developing substrates for SERS [79,80,81,82]. Thermo Fisher proposes a solution for SERS technology dedicated to the DXR3 Raman family of bench-top Raman spectrometers, without indicating that it would check substrates in the context of explosives detection with other portable Raman spectrometers. In addition, Thermo Fisher offers an enhanced hardware and software extension (lowDoseID) for the Gemini device, which enables the detection of trace amounts of key narcotics, including fentanyl, heroin, phenethylamines, synthetic cannabinoids, and cathinones, using the SERS method [83,84]. More details are provided in Section 9. Other commercially available SERS substrates include SERSitive, OceanOptics, Nikalyte, or Hamamatsu Photonics [85,86,87,88]. The use of SERS substrates for explosives detection has a particular advantage due to the sensitivity of explosives. Explosive materials are susceptible to external stimuli, such as mechanical impacts, friction, or vibrations, which can result in unintended detonation. Using a laser in the Raman technique carries a potential risk of initiating the sample of explosive material under analysis. SERS substrates increase detection sensitivity, enabling the use of reduced sample mass or volume, which in turn enhances the safety of the operator performing the analysis. Notably, Heleg-Shabtai et al. demonstrated the practical application of SERS for detecting explosive vapors and particles using a portable Raman spectrometer. In their study, gold nanoparticles deposited on quartz fibers and polyurethane sponges were employed as SERS substrates, enabling the detection of multiple explosives, as exemplified by the detection of pentaerythritol tetranitrate (PETN) at concentrations as low as 1.5 × 10^−6^ M (corresponding to ~6.9 ng) and 2,4,6-trinitrotoluene (TNT) at 1.1 × 10^−7^ M (~0.35 ng) using minimal sample quantities. This approach highlights the feasibility of field-deployable SERS-based detection, while simultaneously reducing the sample mass required and minimizing the risk of unintended initiation of explosive materials [89].

## 4. Fourier Transform Infrared Spectroscopy (FTIR)

In recent years, intensive development of portable spectroscopic systems has been observed to detect explosives in field conditions.

Fourier-transform infrared spectroscopy (FTIR) uses electromagnetic radiation in the 400–4000 cm^−1^ range and is a complementary technique to Raman spectroscopy.

Infrared spectroscopy relies on the absorption of radiation by molecules, which excites characteristic vibrational modes of chemical bonds. Each functional group exhibits absorption within a specific frequency range, producing a unique vibrational fingerprint. By employing a Fourier interferometer, Fourier Transform Infrared Spectroscopy (FTIR) enables simultaneous acquisition of broad spectral ranges, improving speed, coverage, and signal-to-noise ratio compared with conventional scanning methods. Chemical identification is achieved by matching spectral features with reference data. At the same time, preprocessing steps such as background correction, normalization, and multivariate analysis are often required to enhance accuracy under real sample conditions.

Explosive detectors are most commonly developed in the mid-infrared (MIR, ~2.5–25 µm) region, particularly between 6 and 11 µm, where the characteristic vibrational bands of nitro groups (-NO_2_) in compounds such as TNT, RDX, or PETN are strongly absorbed, enabling reliable identification even at trace levels. Near-infrared (NIR, ~0.8–2.5 µm) is also occasionally used in portable systems, but due to weaker absorption bands, multivariate data analysis is often required to achieve accurate differentiation.

As an example, research studies using mid-infrared (mid-IR) approaches, including standoff FTIR systems, have been implemented, enabling the detection of trace amounts of explosives on surfaces such as fabrics or skin, even from distances of several meters. It has been demonstrated that the characteristic vibrational bands of nitro groups present in TNT, RDX, or PETN can be identified despite background interference and variable environmental conditions [90]. In parallel, near-infrared (NIR) systems have been developed, which, when combined with multivariate data analysis, allow for rapid and non-destructive identification of intact explosive charges. Classification algorithms are applied to improve selectivity and to reduce the influence of disturbances resulting from measurement geometry or substrate effects [91]. Both approaches indicate that integrating sensitive spectroscopic techniques with compact portable equipment and advanced signal processing is setting the direction for the further development of field-deployable explosive detectors.

FTIR spectrometers are the third most common mobile detectors for detecting explosives. Table 3 presents eight FTIR spectrometers representing the solutions proposed by leading manufacturers. Operational parameters of detectors, like infrared range and resolution, are similar. Commercially available FTIR detectors are of various designs observed with varying degrees of miniaturization. They can be used to examine gaseous, liquid, or solid samples.

Thermo Fisher offers the most handy solutions for its two most popular detectors, the TruDefender FT and TruDefender FTX, and the analytical capabilities of both models are similar. However, these detectors differ in their construction; the FTX model is a newer, upgraded version of the FT model. The TruDefender FTX model was developed for uniformed services and conformed to military requirements according to MIL-STD 810H. The advantage is the ease of use of the detectors, which require no calibration, heating, or mirror adjustments and have no consumable parts. An important distinguishing feature of this detector is its ability to be decontaminated by the operator after the analysis is complete. The TruDefender FTX can be dipped in a decontaminant or other cleaning agent, then dried, and the device is reused.

Another FTIR spectrometer that complies with the MIL-STD 810H standard is Smith’s Detection HazMatID Elite. The device is characterized by increased weight and reduced portability but offers capabilities similar to the TruDefender FTX regarding the detection range of hazardous substances. The manufacturer declares that it can be used in harsh conditions and high temperatures and can be decontaminated only by immersion.

**Figure 3:**
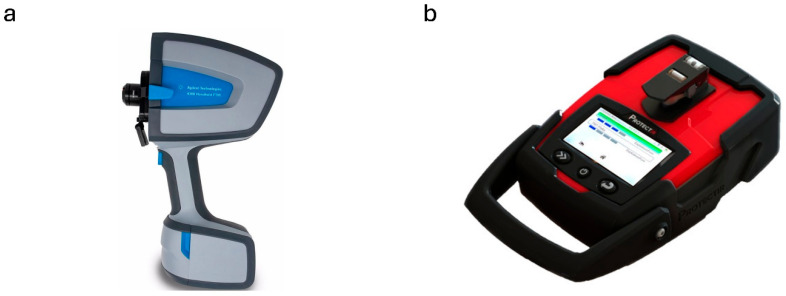
(**a**) 4300 Handheld FTIR Spectrometer from Agilent Technologies [92]; (**b**) ProtectIR from RedWave Technology [94].

Similar class detectors include the 4300 Handheld FTIR Spectrometer from Agilent Technologies (Figure 3a) and ProtectIR from RedWave Technology (Figure 3b). Agilent Technologies does not indicate decontamination rules. Nevertheless, the ProtectIR specification notes an IP67 (Ingress Protection) rating for meeting and compliance with MIL-STD 810H. The IP67 rating defined by EN/IEC 60529 indicates complete dustproofing and protection against immersion to a depth of 1 m. Therefore, it can be assumed that immersing the ProtestIR detector in a decontamination agent will not damage the device.

Agilent Technologies offers portable and suitcase solutions with its 5500 Series Compact FTIR and 4500 Series Portable FTIR models. These devices are small portable platforms designed to perform analysis in non-laboratory conditions. Therefore, the design of these detectors excludes their use by first responders, where analysis time and ease of handling are crucial. However, these detectors could be used, for example, in mobile laboratories.

The suitcase version in its portfolio is also available from RedWave Technology, the ThredID detector. It allows gaseous, liquid, and solid samples to be tested at the ppm (parts per million) level.

## 5. Mass Spectrometry

**Figure 4:**
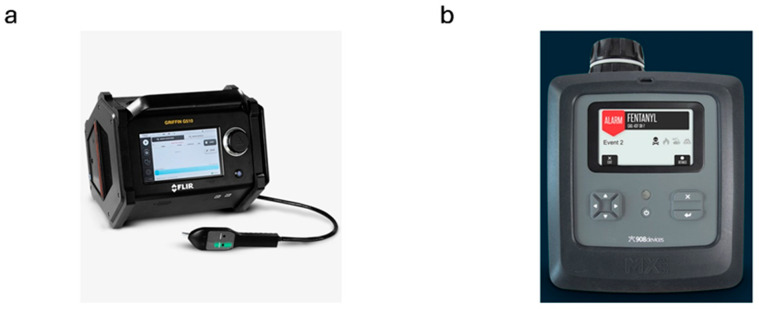
Mass Spectrometers: (**a**) Griffin G510 from Teledyne FLIR [101]; (**b**) MX908 detector from 908 Devices [103].

Mass spectrometry is often paired with gas chromatography, providing powerful analytical capabilities by enabling the separation of mixture components and their detailed identification based on the obtained mass spectra. In recent years, the advantages of combining these two techniques have contributed to an attempt to develop a portable device that gives similar detection capabilities to stationary GC-MS devices. The market offerings for GC-MS are limited compared to mobile detectors based on Raman or FTIR spectroscopy. Table 4 presents commercially available mobile GC-MS instruments equipped with explosives detection capabilities. The Griffin G510 from Teledyne FLIR (Figure 4a) stands out from other detectors. This is attributed to the specific design features of the detector. The device can analyze samples in all states of matter. The Griffin G510 has a heated probe dedicated to gas detection. In addition, using a split/splitless injector enables the direct injection of a liquid sample. The Griffin 510 uses a quadrupole mass spectrometer that allows detection over a mass range of 15–515 m/z with a detection limit of ppb for gases and aerosols and nanograms for liquid and solid samples.

The ion trap mass spectrometer was used in the two mobile devices: Torion T-9 and Portability™ Transportable Mass Spectrometer. The Torion T-9 is coupled to the GC, and the PortabilityTM is a stand-alone mass detector. Torion T-9 uses solid-phase microextraction (SPME) fibers, capillary microextraction, or needle trap as a sample introduction system. Sample dosing methods for GC-MS in identifying explosives and the volatile organic compounds accompanying them were described by S. F. Gallegos et al. [100].

Another type of mobile mass spectrometer is a device that uses a time-of-flight analyzer, such as the Kore MS-200 offered by Kore Technology. The spectrometer detects gases and vapors of hazardous substances, but it is intended for use in industry rather than as a detector in specialized military units. The class of these detectors is characterized by increased mass. Still, the compensation can be an increased range of masses of detected chemical molecules in the range of 1–1000 (m/z) and the detection limit at the ppb level.

High-pressure mass spectrometry is used in the MX908 detector offered by 908 Devices (Figure 4b). According to the supplier, the physical state of the sample does not matter. Gas/aerosols can be analyzed continuously. The manufacturer includes swabbing papers for liquid and solid samples. The MX908 detects explosives at the ppb level. The construction of the device meets the military requirements of MIL-STD-810H.

**Table 4:** Summary of the most important operational parameters of mobile MS detectors.

Manufacturer/Detector Name.	Detectable Substances	The Physical State of the Sample	Limit of Detection/Mass Range	Weight of the Device	References
Teledyne FLIR Detection, Inc./Griffin™ G510	Explosives, Chemical Agents, ITF-25 TIC/TIMs, Narcotics, Other Chemical Targets	Vapor/Gas, Liquid, Solid	ppm, ppb, ng 15–515 m/z	16.3 kg	[101]
Kore Technology Ltd./Kore MS-200	Explosives, Chemical Agents, ITF-25 TIC/TIMs, Narcotics, Other Chemical Targets	Vapor/Gas	ppb 1–1000 m/z	23.0 kg	[102]
908 Devices, Inc./MX908	Explosives (ETN, HMTD, HMX, MEKP, PETN, RDX, TNT, TATP, nitrobenzene and more), Chemical Agents (A-series (Novichoks), HD, GA, GB, GD, GF, VX plus additional V-series agents.), ITF-25 TIC/TIMs (DEEP, DIMP, TEP, GD acid, TMP, dimethyl sulfate, carbon disulfide, allyl amine, allyl alcohol), Narcotics (Fentanyl, fentanyl analogs, U-47700, hydrocodone, oxycodone, heroin, cocaine, amphetamines, ephedrine, MDMA, methadone, methamphetamine, carfentanil, sufentanil, alfentanil, remifentanil, ketamine and more.) Other Chemical Targets (Solid organic materials)	Vapor/Gas, Liquid, Solid	ppb, ng, µg	4.3 kg	[103]
Bay Spec/Portability™ Transportable Mass Spectrometer	Explosives, CWAs, TICs, Pesticides, Narcotics	Aerosol/Gaz/Ciecz Ciało stałe	ppt 50–600 amu 0.49 amu	10.0 kg	[104]
Perkin Elmer/Torion T-9	Explosives (most organic explosives), Chemical Agents, ITF-25 TIC/TIMs, Narcotics, and Other Chemical Targets	Vapor/Gas, Liquid, Solid	ppb 41–500 m/z	14.5 kg	[105]

## 6. Laser Induced Fluorescence (LIF)

Fluorescent photoelectric detection is a spectroscopic technique in which laser illumination induces fluorescence in a sample. In explosive detection, this method is valued for its high sensitivity, selectivity, and capability to perform real-time analyses.

The phenomenon of fluorescence in explosives detection has been used for a long time. Lu Li et al. did a comprehensive review of sensory fluorescent materials regarding the current State-of-the-Art in explosives detection [106]. The authors of the publication point to the construction of mobile devices based on the fluorescence phenomenon as one of the future directions of the development of portable explosives detectors.

Fluorescent photoelectric detection is a spectroscopic technique in which laser illumination induces fluorescence in a sample. In explosive detection, this method is valued for its high sensitivity, selectivity, and capability to perform real-time analyses.

In the study by Weize Shi and Yabin Wang, the application of fluorescent photoelectric detection for identifying organic peroxides, components of many explosives, was presented. It was demonstrated that these substances can be detected at nanomolar levels, making the method promising for security and environmental monitoring. Furthermore, the analysis of samples can be performed in their natural state without the need for complex preparation procedures [107].

The technique’s advantages also include the selective detection of specific chemical groups, which is essential given the chemical diversity of explosive materials. Fluorescent photoelectric detection is applied not only in explosive detection but also in environmental analysis, chemical substance identification, and air quality monitoring.

The findings suggest that the method can be employed effectively in early-warning systems and mobile detection devices, providing high precision and rapid analysis.

**Figure 5:**
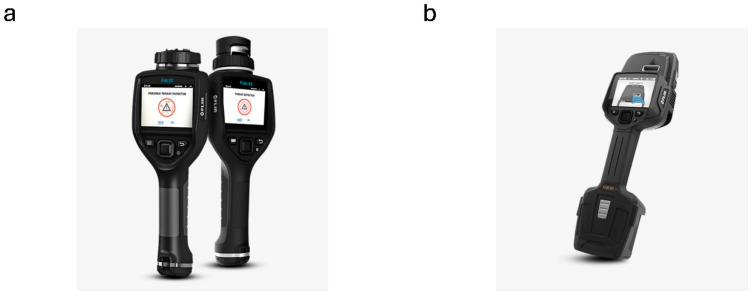
(**a**) FodoX2 [108]; (**b**) FidoX4 from Teledyne FLIR [109].

Teledyne FLIR Detection offers a portfolio of mobile detectors using laser-induced fluorescence (LIF). The FidoX2 and FidoX4 devices are dedicated to identifying explosives (Figure 5 and Table 5).

The result is obtained in up to 10 s, and the lower detection limit for both models is declared at the nanogram level. The manufacturer declares the reusability of the sampling strips. The design of the FidoX4 meets the requirements of MIL-STD-810H.

## 7. Quartz Crystal Microbalance (QCM)

In recent years, there has been a growing interest in using quartz crystal microbalance (QCM) based sensors as a new trend of devices for detecting hazardous materials [110].

Quartz Crystal Microbalance (QCM) is an analytical technique that measures mass changes on a sensor surface by monitoring shifts in the resonance frequency of a quartz crystal. This method has been widely applied for detecting trace amounts of gases and vapors, including explosives. For example, in the study by Procek et al. [111], a QCM sensor coated with TiO_2_ nanostructures was developed to detect nitrogen dioxide (NO_2_) and explosive vapors in air. The sensor demonstrated high sensitivity and selectivity, with frequency changes directly correlated to target molecules’ adsorption on the crystal surface.

The advantages of QCM include real-time monitoring, label-free detection, and the ability to analyze samples without complex pre-treatment. Additionally, the surface functionalization with nanomaterials, such as TiO_2_, enhances the adsorption efficiency and improves the detection limits for trace analytes. The study highlighted that QCM sensors could be effectively utilized in environmental monitoring and security applications, providing rapid and precise detection of hazardous substances.

Detection is based on changing the oscillator frequency when a chemical compound appears in the air being analyzed. MS Technologies is currently developing the method.

Table 6 presents commercially available mobile detectors using the QCM technique that can detect explosives.

**Figure 6:**
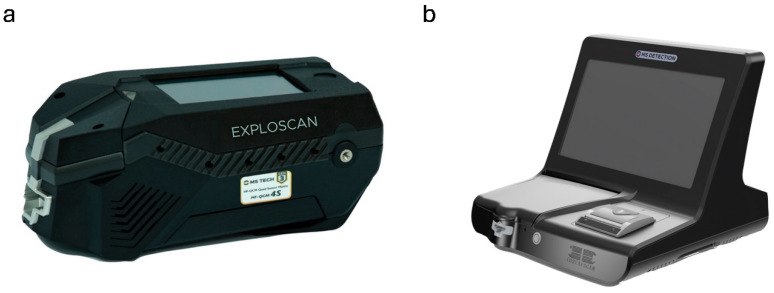
Mobile detectors based on QCM technology from MS Technologies: (**a**) Exploscan [112]; (**b**) Threatscan [114].

Available in two versions of this detector, the civilian and military, Exploscan (Figure 6a) is a device that detects military, plastic, and improvised explosives. The advantage of Exploscan is that there is no carrier gas or radiation source. Exploscan, working in vapor sampling mode, actively draws in air or vapors from the environment using its internal pump. As the air flows through the detection system, the detector analyzes the chemical composition of the vapors. Exploscan indicates whether explosive residues were detected, providing a simple yes/no result or a more detailed identification of the specific substances found. In addition, it is possible to collect a sample from a surface using the included swabs. The swab wipes the surface to gather the sample for later analysis. The swabs are specially designed to collect residues of potential explosives effectively. Analysis time is about 7–15 s, and the detection limit for liquid and solid samples is at the nanogram level and ppb for vapor.

The Duoscan and Multiscan feature an extended detection range for hazardous substances and explosives. The Liquidscan detector, by contrast, is specifically designed to detect liquid explosives and their precursors. The last detector from MS Technologies using QCM technology is ThreatScan, which offers similar detection capabilities but features a slightly different design and increased weight compared to the detectors mentioned above.

## 8. Colorimetric Detection of Explosives

Colorimetric tests use a characteristic chemical reaction involving a color change when the chemical or group of compounds being determined comes into contact with the chemical reagents present in a colorimetric test.

Colorimetric detection methods for explosives continue to be actively researched and applied due to their simplicity, rapid response, and cost-effectiveness. Recent studies have focused on enhancing these methods’ sensitivity, selectivity, and practicality for field applications.

A 2015 study by Idros et al. introduced a colorimetric detection system for TNT based on functionalized silica nanoparticles. The primary amine groups on the nanoparticles facilitated the formation of Meisenheimer complexes with TNT, resulting in a visible color change. This label-free and selective detection method provided a straightforward and efficient means for TNT identification [115].

Continuing these efforts in a 2021 study by Thipwimonmas et al., a digital image-based colorimetric method was developed utilizing polymer gel sensors on 96-well plates. This approach demonstrated high sensitivity and specificity for detecting multiple explosives, including TNT, DNT, nitrite, and perchlorate. The method’s effectiveness was validated through RGB color analysis of reaction products, offering a reliable tool for on-site screening of explosive traces [116].

These advancements underscore the ongoing relevance and development of colorimetric detection techniques in explosive trace detection, highlighting their potential for integration into portable and user-friendly detection systems.

A wide range of color tests indicating the presence of explosives is commercially available. The operator interprets the color change based on the color templates most often included in the instructions supplied with the test kit. The user’s reading of color can be highly subjective and is affected by visual defects, indication reading conditions, light intensity, operator exhaustion, and analyte concentration that is too low. Commercially available are electronic readers, so-called colorimeters, which allow digital objectification of the color obtained, reducing the possibility of operator error. In most cases, the detection limit of color tests is ppm, µg, and, less often, ng.

### 8.1. Colorimetric Detection of Explosives Interpreted by the Operator

**Figure 7:**
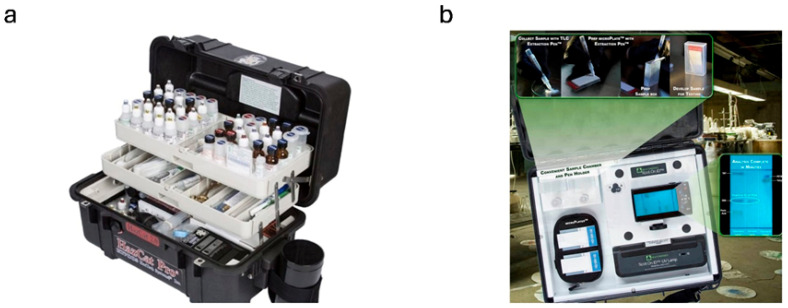
(**a**) Test kit HazCat 2.0 Pro from Haztech Systems [121]; (**b**) Spot. On.ID kit from Field Forensics [123].

Examples of colorimetric tests for detecting explosives, commercially available solutions are shown in Table 7. Due to the wide range of available colorimetric tests, presenting representative examples of solutions designed to indicate the presence of explosives was chosen as the criterion for the compilation. In general, commercially available color tests mostly consist of kits of paper/swabs onto which a sample of potentially hazardous material is collected. Visualization can occur by spraying with an aerosol, applying a drop of the appropriate reagent, or immersing in the finished solution. Other examples of colorimetric tests include indicator tubes. A test involves collecting a specific amount of gas and passing it through a tube, which is facilitated by a pump. The tube contains carefully selected chemical reagents that change color in response to explosives. Kits containing various types of colorimetric tests are available for detecting hazardous materials, including explosives. An example of such a test kit is HazCat 2.0 Pro, offered by Haztech Systems Inc. (Figure 7a), designed for use by first responders in the field. The HazCat 2.0 Pro includes comprehensive colorimetric tests and detailed charts outlining the steps for operators to identify explosive materials.

The TLC thin-layer chromatography technique is used in the Spot. On.ID kit (Figure 7b) is usable in field and laboratory conditions. It enables the separation and identification of substances that colorimetric tests cannot detect. This solution is an alternative between simple color tests and analytical techniques. The nanogram-level detection of explosives and the fact that the plates and sampler (the Spotter) can be given additional analysis in the laboratory are undoubted advantages of the Spot. On.ID kit.

### 8.2. Colorimeters

**Figure 8:**
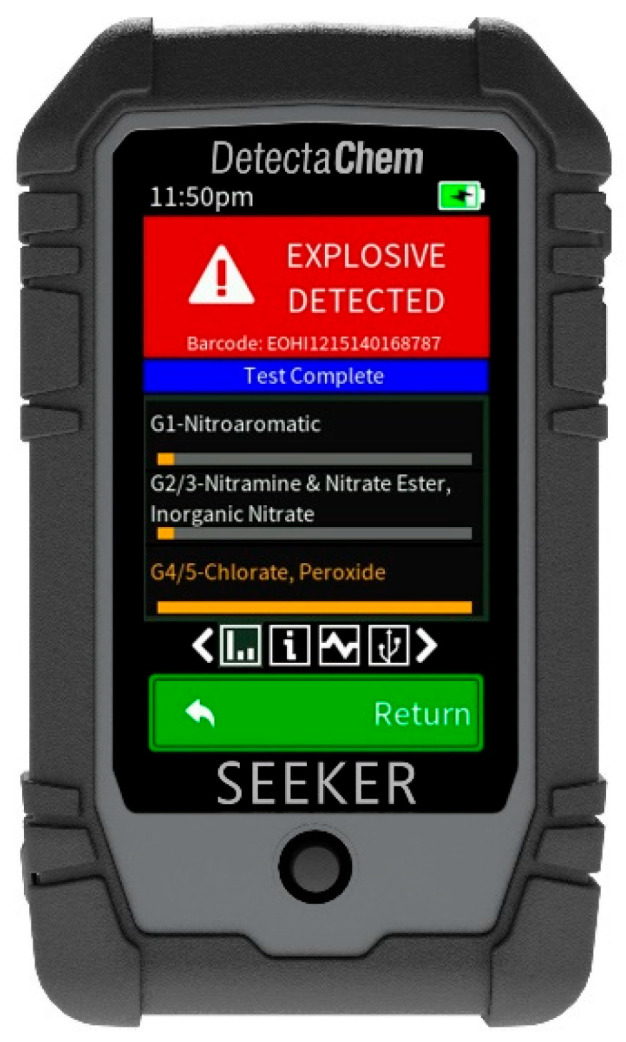
Colorimeter SEEKERe from DetectaChem [124].

Colorimeters help detect explosives by measuring the change in color of a chemical reagent after it reacts with explosive compounds. The colorimeter shines light through the sample and measures the intensity of the transmitted or reflected light at specific wavelengths. Using a colorimeter to read the color change of a test avoids the error of interpreting the result. Table 8 presents a representative example of such a device, the SEEKERe device from DetectaChem (Figure 8).

A sample is collected using a specially designed test card containing specific reagents housed in microfluidic chambers. The test card is inserted into the SEEKERe device, where the reagents react with the sample. These reactions are designed to produce a distinct color change if explosives are present. The SEEKERe device employs its optical sensors to detect and analyze the color changes on the test card. Embedded algorithms process this data to determine the chemical composition of the sample. The results are displayed on the device’s screen. Typically, the results are generated within a few seconds.

## 9. Coupling Raman Spectroscopy with FTIR

**Figure 9:**
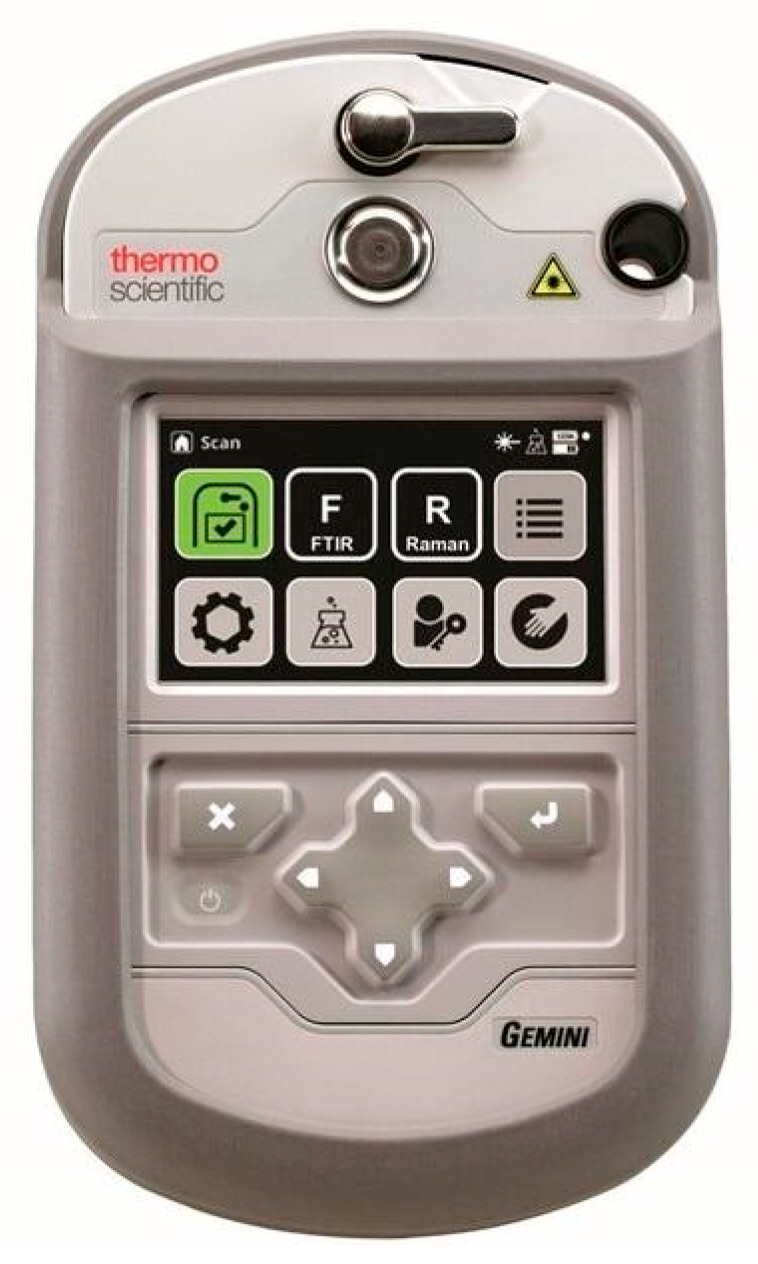
Detector Gemini from Thermo Fisher [125].

A unique mobile device called Gemini (Figure 9 and Table 9), which enables the analysis of samples using two complementary analytical techniques, i.e., Raman and FTIR spectroscopy, has been developed by Thermo Fisher. The detector is equipped with a probe on a flexible arm to ensure that testing can be conducted in hard-to-reach places without moving the sample. Gemini’s design complies with MIL-STD-810H, and it is possible, as with the TruDefender FTX detector, to decontaminate it by immersing the detector in a decontamination agent.

Gemini’s light weight allows for easy analysis in a potentially contaminated zone. The producer declares that the touchscreen has a high sensitivity for gloved operation, and as an option, there is a simplified keyboard control. Gemini has two LowDoseID software extensions that, together with SERS analysis accessories, allow for increased detection sensitivity for Raman spectroscopy. Gemini includes HazMasterG3, an advanced software that enhances its chemical and hazardous material identification capabilities. HazMasterG3 can perform sophisticated scenario analyses, providing insights into potential reactions or threats posed by identified substances. For example, it can simulate the outcomes of mixing specific chemicals or precursors and evaluate the risks associated with identified substances. The software delivers actionable guidance directly on-screen, enabling users to respond quickly and effectively. The HazMasterG3 add-on transforms the Gemini detector into a comprehensive chemical analysis and hazard assessment tool. The supplement HazMasterG3 was developed mainly for personnel of chemical and pyrotechnic rescue groups.

**Figure 10:**
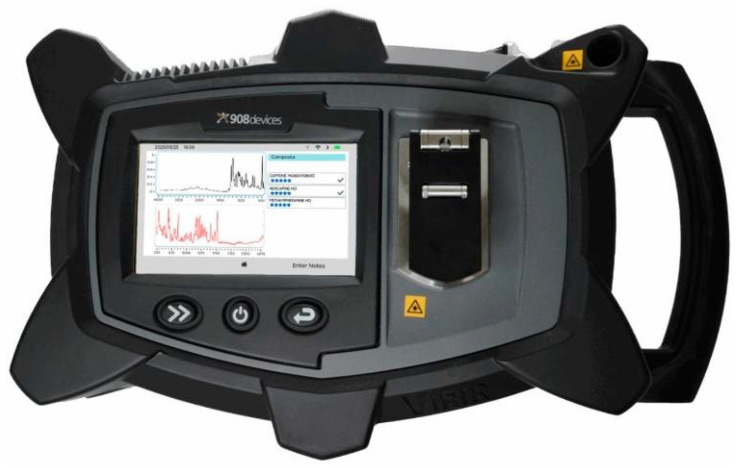
FTIR/Raman detector VipoIR from 908devices [126].

One of the most recent and technologically advanced field-deployable explosive detectors developed by 908 Devices is the VipIR portable spectrometer (Figure 10 and Table 9), explicitly designed for customs, border security, CBRN response teams, and explosive ordnance disposal units. Unlike earlier generations of portable Raman or FTIR instruments, which required the operator to decide which spectroscopic technique was most appropriate for a given sample, VipIR integrates mid-infrared Fourier Transform Infrared (FTIR) spectroscopy and Raman spectroscopy within a single platform, enhanced by a proprietary algorithm known as Smart Spectral Processing (SSP). This innovation allows the system to perform simultaneous analyses using both methods on a single sample and deliver one unified, high-confidence result. For operators in the field, this means faster decision-making and a substantial reduction in the risk of ambiguous or contradictory identifications. The system has a comprehensive reference library of more than 39,000 spectra, including narcotics, explosives, chemical warfare agents (CWAs), toxic industrial chemicals (TICs/TIMs), and consumer products.

The instrument includes a flexible Raman probe for through-container analysis, a sample vial holder, and support for Surface-Enhanced Raman Spectroscopy (SERS), which extends detection capabilities to trace levels of explosive materials or explosive precursors. VipIR’s ability to recognize complex mixtures and report multiple observable components is critical when analyzing impure or deliberately masked explosive formulations. The device is engineered for demanding operational environments and is rugged to IP67 and MIL-STD-810G standards, ensuring water, dust, and shock resistance. The five-inch touch display, connectivity options (Wi-Fi and LTE), and the dedicated Team Leader application for result sharing and remote fleet management underscore its ergonomic and network-ready design, tailored to multi-agency and distributed operations. For explosive detection specifically, VipIR’s dual-technology approach offers several operational strengths. First, integrating FTIR and Raman reduces false positives and negatives by cross-validating spectral signatures. Second, the device’s advanced mixture analysis capability is particularly valuable for detecting improvised explosive formulations, which often combine multiple organic and inorganic components. Third, SERS support extends the instrument’s functionality to trace analysis, which is essential for forensic applications and the identification of low concentrations of energetic compounds. Nevertheless, limitations remain: FTIR and Raman are not inherently ultra-trace sensitive without SERS enhancement.

## 10. IMS Coupling with an Electrochemical Sensor

**Figure 11:**
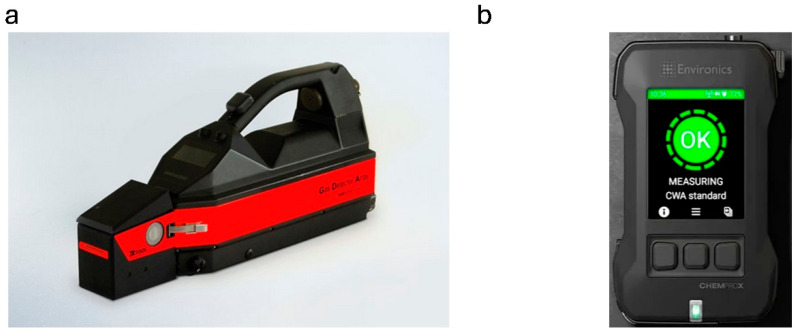
(**a**) GDP-X detector from Airsense Analytics [128]; (**b**) ChemProX detector from Environics [129].

Detectors utilizing multiple analytical techniques enable the analysis of a broader range of hazardous substances, enhancing the reliability of the results. Table 10 presents commercially available portable detectors with such solutions. A unique solution, utilizing the GDP-X detector (Figure 11a), is provided by Airsense Analytics, which enables the analysis of substances with low vapor pressure, including many explosives. For this purpose, an X-TOOL attachment is mounted to the front of the device, where the substance is desorbed into a gaseous state. Additionally, it is stated that a Geiger–Muller sensor can be integrated, allowing the device’s operational range to be extended to include the detection of gamma and X-rays.

Another example of using IMS technology with a solid-state sensor is the ChemProX detector, which was also made to meet military strength standards (Figure 11b). ChemProX has various sensors that analyze air samples in real time. It is designed with mobility and ease of use in mind, allowing for rapid response in emergencies. ChemProX also offers the capability to network with other devices, enabling coordinated efforts within larger teams and enhancing operational efficiency when managing chemical threats.

## 11. Conclusions

In recent years, detecting explosives outside specialized laboratories has become increasingly critical, particularly in light of current global events. With growing security concerns and the rising threat of terrorism, the need for portable and rapid detection systems has never been more urgent. The ability to perform these analyses in the field, rather than relying solely on laboratory-based tests, is crucial for ensuring public safety and addressing emerging threats in real time. Therefore, the issue of developing better and better field detectors for detecting explosives is still topical. Market analysis reveals a wide range of explosive detectors featuring diverse design solutions and varying levels of complexity. This trend is a direct result of tremendous technological advances in microelectronics and the ability to incorporate specialized software into detectors. One of the most important indicators of the development potential of a technique/detector is the number of models from different producers. According to this criterion, ion mobility spectrometry (IMS) is a technique with high potential. The second such technique is Raman spectroscopy. These techniques have the unquestionable advantage of enabling analysis without physical contact with the sample. It is worth noting that the review identified approximately 80 field devices; however, it did not encompass all detectors, as many are produced in limited series or even as single units for domestic markets. This suggests that certain developments may be emerging at an early stage, remaining undetected on the international scale.

As an example, one can refer to the detector developed by M. Maziejuk, T. Sikora, and W. Lisowski at the Military Institute of Chemistry and Radiometry in Warsaw. This detector employs differential ion mobility spectrometry and ion mobility spectrometry (DMS/IMS) for the detection of volatile organic compounds. This approach allows for sensitive, non-contact detection of multiple analytes at trace levels, demonstrating how advanced detection technologies can be developed in limited series while remaining highly effective [130].

These countries produce a variety of detectors, but very often, they are subject to export bans.

An attempt to predict the most significant trends in development leads to the conclusion that manufacturers will continue to strive for the miniaturization of detectors, the integration of multiple analytical techniques in a single detector, and, as is already becoming apparent, reducing operator involvement to a minimum during the detection process. The first significant development path for field detectors is the coupling of techniques. Dual-technology detection systems significantly improve over single-technology devices, reducing false alarm rates from 10–15% to below 2%, while incurring an approximate 40% increase in weight. This approach demonstrates enhanced accuracy and reliability, indicating that future development should focus on optimizing the trade-off between performance and portability. Flagship examples in this category are the Gemini (Thermo Fisher), VipIR (908devices), GDA-X (Airsense), or Griffin 510 (FLIR) detectors.

The development prospects of VipIR and similar devices lie in several areas. The fusion of multiple spectroscopic datasets through SSP represents an essential step toward intelligent sensor integration, and future versions are expected to incorporate more advanced machine learning techniques for automated quality assessment, mixture deconvolution, and confidence scoring. The standardization and broader availability of robust SERS substrates will further enhance trace detection capabilities in the field, reducing operator dependency. Continued miniaturization may lower the weight and size of such instruments, making them more suitable for dismounted patrols, while expanded cloud connectivity will enable centralized updates to spectral libraries, real-time collaboration, and improved incident tracking across agencies. Finally, interoperability with other detection technologies (IMS, GC-MS, or particle detectors) will likely become a defining feature of next-generation field detection systems, providing security forces and first responders with a more complete operational awareness.

A second development path involves using new techniques that have not yet been commercialized, such as Thermally Enhanced Fourier-Transform Infrared Spectroscopy (TE-FTIR). The profiles of gaseous decomposition products, explosives, and their temperature-dependent changes, recorded with TE-FTIR, are distinctive and can be used to identify explosives based on their decomposition products [131]. Another example is the creation of electrodes that are selective toward specific hazardous compounds, which are then used in voltammetry and electrochemical impedance spectroscopy [132].

A third path forward is using analytical techniques not previously considered in portable or field detectors. An excellent example is the described series of detectors from MS Technologies that use a quartz microbalance. Although the method has been known for years, it is only recently that an attempt has been made to use it in field detectors.

During field detection work, the device’s physical durability and ergonomics are crucial. The device must be waterproof, dustproof, and shockproof, among other necessities. It is also crucial that the device can be operated by an operator wearing thick protective gloves. The solutions used in the VipIR are noteworthy: the device is equipped with comfortable grips and large control buttons. Similar design solutions were used in the Gemini device and AP4C, where the number of activities to be performed by the operator is reduced to a minimum.

## Figures and Tables

**Table 1:** A summary of detectors’ most critical performance parameters using the ion mobility spectrometry (IMS) technique.

Manufacturer/Detector Name.	Detectable Substances	The Physical State of the Sample	Detection Limit	Weight of the Device	References
Seer Technology Inc./Accu Sense	Explosives, Narcotics, Chemical Agents, ITF-25 TIC/TIMs, Other Chemical Targets	Vapor/Gas	ppm, ppb	10.4 kg	[19,20,21]
Scintrex Trace Corp./E3500Trace Detector	Explosives (TNT, RDX, NG, AN, PETN, EGDN, HMX, and UN), Narcotics (Cocaine, opiates, THC, heroin, amphetamine-type stimulants, fentanyl, carfentanil, sufentanil, alfentanil, remifentanil, ketamine, lorazepam, 3-quinuclidinyl benzilate, U-47700)	Vapor/Gas/Solid	ng	3.7 kg	[22]
Leidos/QS-H150 H150E	Explosives, ITF-25 TIC/TIMs, Narcotics	Vapor/Gas, Liquid, Solid	ng	5.0 kg	[23,24]
Smith’s Detection, Inc./LCD 4 XID	Explosives, CWA, TIC, Narcotics	Vapor/Gas	ppm	0.58 kg	[25]
Scintrex Trace Corp./N2300 Trace Detector	Explosives (TNT, RDX, NG, AN, PETN, EGDN, HMX, and UN), Narcotics (Cocaine, opiates, THC, heroin, amphetamine-type stimulants, fentanyl, carfentanil, sufentanil, alfentanil, remifentanil, ketamine, lorazepam, 3-quinuclidinyl benzilate, U-47700)	Vapor/Gas	ng	3.2 kg	[26]
Bruker Corporation/RoadRunner	Explosives (Pentaerythritol tetranitrate (PETN), Cyclotrimethylenetrinitramine/Hexogen (RDX), Trinitrotoluene (TNT), Ammonium Nitrate (NIT), Urea Nitrate (NIT), Triacetone triperoxide (TATP), Hexamethylene triperoxide diamine (HMTD), Nitroglycerine (NG), 2,4,6-Trinitrophenylmethylnitramine/Tetryl (TET), 2,3-dimethyl-2,3-dinitrobutane (DMNB), Black powder (BP), Ethylene glycol dinitrate (EGDN), 2,4-Dinitrotoluene (DNT), Narcotics (Cocaine (COC), Methamphetamine (METH), 3,6-Diacetylmorphine (Heroin) (HER), delta9-Tetrahydrocannabinol (THC), 3,4-Methylenedioxyamphetamine	Vapor/Gas, Solid	µg	3.5 kg	[27]
Security Electronic Equipment Co., Ltd./SPE300	Explosives, Chemical Agents, ITF-25 TIC/TIMs, Narcotics, Other Chemical Targets (Sulfur)	Vapor/Gas, Liquid, Solid	ng, pg	4.3 kg	[28]
Nuctech Company Limited/TR1000DB-A	Explosives, Narcotics	Vapor/Gas, Liquid, Solid	ng	4.0 kg	[29]
Smith’s Detection, Inc./Ionscan 600	Explosives, Chemical	Vapor/Gas, Liquid, Solid		10.8 kg (11.5 kg with printer)	[30]
Security Electronic Equipment Co., Ltd./SPE9000 Series	Explosives, Narcotics	Vapor/Gas, Liquid, Solid	ng	3.0 kg	[31]
Owlstone, Inc./Lonestar Portable Analyzer	Explosives, Chemical Agents, ITF-25 TIC/TIMs (DNT, DMNB, RDX, TNT, and MNT)	Vapor/Gas	ppb	7.8 kg	[32]
Inward Detection/M-ION	Explosives (TNT, RDX, PETN, ANFO, EGDN, Nitroglycerine (NG), Dynamite, DNT, Black Powder, Ammonia and Urea Nitrates, HMTD, Tetryl, and mixtures thereof)	Vapor/Gas	ppt	3.0 kg	[33]
Rapiscan Systems/MobileTrace	Explosives, ITF-25 TIC/TIMs, Narcotics	Vapor/Gas	µg	4.3 kg	[34]
Rapiscan Systems/Hardened MobileTrace (HMT)	Explosives, Chemical Agents, ITF-25 TIC/TIMs, Narcotics, Other Chemical Targets	Vapor/Gas	ppb	5.4 kg	[35]
Siebel/MO-2M	TNT, RDX, NG, AN	Vapor/Gas	10^−13^ g/cm^3^	1.5 kg	[36]

**Table 2:** Summary of operational parameters of detectors using Raman spectroscopy.

Manufacturer/Detector Name.	Detectable Substances	The Physical State of the Sample	Range of Electromagnetic Radiation/Length of Laser/Resolution	Weight of the Device	References
Optosky Photonics Inc./ATR6200	Explosives, Chemical Agents, ITF-25 TIC/TIMs, Narcotics, Other Chemical Targets	Liquid, Solid	Laser: 785 nm, 250–2400 cm^−1^ or 200–3300 cm^−1^ (HS), 13–15 cm^−1^ (HS)	1.2 kg	[39]
Optosky Photonics Inc./ATR6500/6600	Explosives, Chemical Agents, ITF-25 TIC/TIMs, Narcotics, Other Chemical Targets	Liquid, Solid	200 to 4000 cm^−1^, 10 cm^−1^/8–12 cm^−1^	0.45 kg/1.15 kg	[40,41]
Bruker Corporation/Bravo	Explosives, Chemical Agents, ITF-25 TIC/TIMs, Narcotics, Other Chemical Targets	Liquid, Solid	3200–300 cm^−1^ 10–12 cm^−1^ Laser: 700–1100 nm	1.5 kg	[42]
Coda Devices, Inc./CDI 2	Explosives, Chemical Agents, ITF-25 TIC/TIMs, Narcotics, Other Chemical Targets	Liquid, Solid	500–1800 cm^−1^ 6–8 cm^−1^	3.0 kg	[43]
Anton Paar USA, Inc./Cora 100	Explosives, Chemical Agents, ITF-25 TIC/TIMs, Narcotics, Other Chemical Targets	Liquid, Solid	Laser 785 nm 400 to 2300 cm^−1^ 10 cm^−1^	0.65 kg	[44]
Rigaku Analytical Devices/CQL Gen-ID	Explosives Precursors, Narcotics, Narcotic Precursors, Pharmaceuticals, Steroids, Other Chemical Targets (Biomolecule (amino acids), Cutting Agents, General Chemicals, General Precursors, Household Chemicals, Industrial Chemicals, Polymers, Solvents)	Liquid, Solid	Laser 1064 nm 200–2500 cm^−1^ 6–13 cm^−1^	1.7 kg	[45]
Rigaku Analytical Devices/CQL Max-ID	Explosives and Precursors Chemical Agents (CWAs (including 4th generation), CWA Precursors), ITF-25 TIC/TIMs, Narcotics and Precursors, Pharmaceuticals, Steroids Other Chemical Targets (Biomolecule (amino acids), Cutting Agents, General Chemicals, General Precursors, Household Chemicals, Industrial Chemicals, Pesticides, Polymers, Solvents)	Liquid, Solid	Laser 1064 nm 200–2500 cm^−1^ 6–13 cm^−1^	1.7 kg	[46]
Thermo Fisher Scientific Inc./FirstDefender RM	Explosives, Chemical Agents, ITF-25 TIC/TIMs, Narcotics, Other Chemical Targets	Liquid, Solid	250–2875 cm^−1^ 7–10.5 cm^−1^	0.8 kg	[47]
Thermo Fisher Scientific Inc./FirstDefender RMX	Explosives, Chemical Agents, ITF-25 TIC/TIMs, Narcotics, Other Chemical Targets	Liquid, Solid	250–2875 cm^−1^ 7 to 10.5 cm^−1^	0.919 kg	[48]
RS DYNAMICS/microRAMAN	Explosives, Chemical Agents, ITF-25 TIC/TIMs, Narcotics, Other Chemical Targets	Liquid, Solid	200–2000 cm^−1^ 12 cm^−1^	0.65 kg	[49]
RS DYNAMICS/miniSPECTRE	Explosives, Chemical Agents, ITF-25 TIC/TIMs, Narcotics, Other Chemical Targets	Liquid, Solid	ng, 400 cm^−1^ to 2300 cm^−1^ 8–10 cm^−1^	0.95 kg	[50]
Metrohm/MIRA DS/XTR DS	Explosives, Chemical Agents, ITF-25 TIC/TIMs, Narcotics, Other Chemical Targets	Liquid, Solid	mg	2.5 kg	[51]
Metrohm/NanoRam	Explosives, Chemical Agents, ITF-25 TIC/TIMs, Narcotics, Other Chemical Targets	Liquid, Solid	mg, Laser 785 nm: 176 cm^−1^ to 2900 cm^−1^ Laser 1064 nm: 176 cm^−1^ to 2500 cm^−1^	1.2 kg	[52]
Pendar Technologies/Pendar X10	Explosives, Chemical Agents, ITF-25 TIC/TIMs, Narcotics, Other Chemical Targets	Liquid, Solid	mg	2.0 kg	[53]
Chemring Detection Systems/PGR-1064	Explosives, Chemical Agents, ITF-25 TIC/TIMs, Narcotics, Other Chemical Targets	Liquid, Solid	mg, 350 cm^−1^ to 1850 cm^−1^ 8 cm^−1^	1.0 kg	[54]
Agiltron/PinPointer	Explosives, Chemical Agents, ITF-25 TIC/TIMs, Narcotics, Other Chemical Targets	Liquid, Solid	200 to 3000 cm^−1^ 9 cm^−1^	1.4 kg	[55]
Rigaku Analytical Devices/Progeny	Explosives, Chemical Agents, ITF-25 TIC/TIMs, Narcotics, Other Chemical Targets	Liquid, Solid	200 to 2500 cm^−1^	1.6 kg	[56]
BioTools/RamTest	Explosives, Chemical Agents, ITF-25 TIC/TIMs, Narcotics, Other Chemical Targets	Liquid, Solid	100–4000 cm^−1^	2.0 kg	[57]
Agilent Technologies/Resolve Handheld SORS	Explosives Chemical Agents, ITF-25 TIC/TIMs, Narcotics (fentanyl, carfentanil, sufentanil, alfentanil, remifentanil, heroin, ketamine, lorazepam, 3-quinuclidinyl benzilate, U-47700) Other Chemical Targets	Liquid, Solid	mg, 350–2000 cm^−1^ 10 cm^−1^	2.2 kg	[58]
NucTech Company Limited/RT6000S	ITF-25 TIC/TIMs, Explosives (TNT, RDX, Nitramine Explosive, C3, HMX, Composition B, TATP, PETN), Narcotics (Ketamine, cocaine, morphine, others amenable based on technology)	Liquid, Solid	Data not available	0.47 kg	[59]
Serstech/Serstech 100 Indicator	Explosives, Chemical Agents, ITF-25 TIC/TIMs, Narcotics, Other Chemical Targets	Liquid, Solid	400 to 2300 cm^−1^	0.65 kg	[60]
StellarNet/StellarRAM	Explosives, Chemical Agents, ITF-25 TIC/TIMs, Narcotics, Other Chemical Targets	Liquid, Solid	200 to 2250 cm^−1^ 12 cm^−1^	2.5 kg	[61]
Metrohm/TacticID Series	Explosives, Chemical Agents, ITF-25 TIC/TIMs, Narcotics, Other Chemical Targets	Liquid, Solid	179 to 2900 cm^−1^ 9 cm^−1^	0.9 kg	[62]
Thermo Fisher Scientific Inc/TruScan RM	Explosives, Chemical Agents, ITF-25 TIC/TIMs, Narcotics, Other Chemical Targets	Liquid, Solid	250 to 2875 cm^−1^ 8–10.5 cm^−1^	0.9 kg	[63]
Agilent Technologies/Vaya	Explosives, Chemical Agents, ITF-25 TIC/TIMs, Narcotics, Other Chemical Targets	Liquid, Solid	350–2000 cm^–1^	1.6 kg	[64]
Horiba Scientific/AnywhereRaman	Explosives, Chemical Agents, ITF-25 TIC/TIMs, Narcotics, Other Chemical Targets	Liquid, Solid	150–3150 cm^−1^ 5–6 cm^−1^	3.6 kg	[65]
Coda Devices Inc./CDI 1M	Explosives, Chemical Agents, ITF-25 TIC/TIMs, Narcotics, Other Chemical Targets	Liquid, Solid	500–1800 cm^−1^ 300–2900 cm^−1^ 6–8 cm^−1^ 8–9 cm^−1^	11.0 kg	[66]
Metrohm/IM-52	Explosives, Chemical Agents, ITF-25 TIC/TIMs, Narcotics, Other Chemical Targets	Liquid, Solid	200–3200 cm^−1^ 4–8 cm^−1^	10.0 kg	[67]
InPhotonics Inc./InPhochelle	Explosives, Chemical Agents, ITF-25 TIC/TIMs, Narcotics, Other Chemical Targets	Liquid, Solid	100–3500 cm^−1^ 2 cm^−1^	7.7 kg	[68]
InPhotonics Inc./InPhotote	Explosives, Chemical Agents, ITF-25 TIC/TIMs, Narcotics, Other Chemical Targets	Liquid, Solid	mg 250–2350 cm^−1^ 2 cm^−1^	10.0 kg	[69]
Metrohm/i-Raman/Raman Prime	Explosives, Chemical Agents, ITF-25 TIC/TIMs, Narcotics, Other Chemical Targets	Liquid, Solid	mg, 150–4000 cm^−1^ 150–3300 cm^−1^ 150–3200 cm^−1^ 150–2700 cm^−1^	3.0 kg	[70]
Optosky Photonics Inc./Portable Raman Analyzer Series	Explosives (50 substances), Chemical Agents, ITF-25 TIC/TIMs, Narcotics (150 substances), Other Chemical Targets (over 50 substances)	Liquid, Solid	mg, 50 cm^−1^ to 4000 cm^−1^ 19 cm^−1^	7.5 kg	[71]
Alakai Defense Systems/PRIED	Explosives, Chemical Agents, ITF-25 TIC/TIMs, Narcotics (Heroin, cocaine, methamphetamine, fentanyl), Other Chemical Targets (HAZMAT, sugar, sodium bicarbonate, acetaminophen)	Liquid, Solid	mg, 400–2000 cm^−1^ <10 cm^−1^	2.9 kg	[72]
Advanced Nano Technologies/Raman Flipper	Explosives, Chemical Agents, ITF-25 TIC/TIMs, Narcotics, Other Chemical Targets	Liquid, Solid	ng 638 nm: 270–2400 cm^−1^ 785 nm: 270–2000 cm^−1^ 830 nm: 200–1850 cm^−1^ 1064 nm: 250–1850 cm^−1^ 6 cm^−1^	7.1 kg	[73]
Agilent Technologies/RapID	Explosives, Chemical Agents, ITF-25 TIC/TIMs, Narcotics, Other Chemical Targets	Liquid, Solid	~10%	47.0 kg	[74]
StellarNet/StellarCASE-Raman	Explosives, Chemical Agents, ITF-25 TIC/TIMs, Narcotics, Other Chemical Targets	Liquid, Solid	200–2300 cm^−1^ 4 cm^−1^	5.4 kg	[75]

**Table 3:** Summary of the most important parameters of the performance of mobile FTIR spectrometers.

Manufacturer/Detector Name.	Detectable Substances	The Physical State of the Sample	Infrared Radiation Spectrum/Resolution/Limit of Detection	Weight of the Device	References
Agilent Technologies/4300 Handheld FTIR Spectrometer	Explosives, Chemical Agents, ITF-25 TIC/TIMs, Narcotics. Other Chemical Targets	Liquid, Solid	5200 to 650 cm^−1^ 4 to 16 cm^−1^	2.2 kg	[92]
Smith’s Detection, Inc./HazMatID Elite	Explosives, Chemical	Liquid, Solid	4000–650 cm^−1^ 100 ppm- 1 ppt	2.29 kg	[93]
908devices/ProtectIR	Explosives, Chemical Agents, ITF-25 TIC/TIMs, Narcotics. Other Chemical Targets	Liquid, Solid	4000–650 cm^−1^ 4 cm^−1^	2.3 kg	[94]
Thermo Fisher Scientific, Inc./TruDefender FT	Explosives, Chemical Agents (GA, GB, GD, HD, HNs, VX, RVX, others), ITF-25 TIC/TIMs, Narcotics. Other Chemical Targets (All IR-active organic compounds)	Liquid, Solid	4000–650 cm^−1^/ 4 cm^−1^	1.3 kg	[95]
Thermo Fisher Scientific, Inc./TruDefender FTX	Explosives Chemical Agents (GA, GB, GD, HD, HNs, VX, RVX, others), ITF-25 TIC/TIMs, Narcotics. Other Chemical Targets (All IR-active organic compounds)	Liquid, Solid	4000–650 cm^−1^/ 4 cm^−1^	1.41 kg	[96]
Agilent Technologies/4500 Series Portable FTIR	Explosives, Chemical Agents, ITF-25 TIC/TIMs, Narcotics (Cocaine hydrochloride, cocaine base, diacetylmorphine, morphine hydrochloride, ketamine, fentanyl, carfentanil, sufentanil, alfentanil, remifentanil, heroin, ketamine, lorazepam, 3-quinuclidinyl benzilate, U-47700) Other Chemical Targets	Liquid, Solid	4000–650 cm^−1^/ 2 to 32 cm^−1^	6.8 kg	[97]
Agilent Technologies/5500 Series Compact FTIR	Explosives, Chemical Agents, ITF-25 TIC/TIMs, Narcotics. Other Chemical Targets	Liquid, Solid	4000–650 cm^−1^/ 2 to 32 cm^−1^	3.6 kg	[98]
908devices/ThreatID	Explosives, CWAs, Hazardous Chemicals (TICs, TIMs, VOCs), Narcotics	Vapor/Gas, Liquid, Solid	25 ppm	6.3 kg	[99]

**Table 5:** Summary of the most important operational parameters of detectors using laser-induced fluorescence.

Manufacturer/Detector Name.	Detectable Substances	The Physical State of the Sample	Limit of Detection	Weight of the Device	References
Teledyne FLIR Detection, Inc/FidoX2	Explosives (three detection targets, military, nitrate, peroxide)	Liquid, Solid	ng	0.7 kg	[108]
Teledyne FLIR Detection, Inc./FidoX4	Explosives (Nitro aromatics, Nitramines, Nitrate-esters, Nitrosamines, Inorganic Nitrates, Plastic Explosives, Smokeless Powders, and Peroxides)	Liquid, Solid	ng	1.5 kg	[109]

**Table 6:** Mobile detectors based on QCM technology from MS Technologies.

Manufacturer/ Detector Name.	Detectable Substances	The Physical State of the Sample	Limit of Detection	Weight of the Device	References
MS Technologies Inc./EXPLOSCAN/ DUOSCAN/ MULTISCAN	Military and plastic explosives, including TNT, Tetryl, RDX, C4, PETN, Semtex, HMX, Datasheet, Dynamite, Nitroglycerine, and others. Peroxide-based explosives, including TATP, HMTD, and others. Nitrate-based explosives include ammonium nitrate, urea nitrate, and others. Propellants and Taggants, including Black and Smokeless Powder, EGDN, and others. Chlorates, Perchlorates, and Sulfur-based HMEs. Heroin, Cocaine, Amphetamine, Methamphetamine, Ketamine, MDA, THC, LSD, Ecstasy, and others. Synthetic Opioids: Fentanyl, Carfentanyl, W-18, and others.	Vapor/Gas, Liquid, Solid	Exploscan: ng, ppb Duoscan/ Multiscan: ng, ppm	0.85 kg	[112]
MS Technologies Inc./LIQUISCAN	Liquid explosives, narcotics, and TIC/TIMs	Vapor/Gas, Liquid, Solid	ng, ppm	0.66 kg	[113]
MS Technologies Inc/THREATSCAN	Military and plastic explosives include TNT, Tetryl, RDX, C4, PETN, Semtex, HMX, Datasheet, PEK, Gelatine, Sheet Explosives, LTPE, and. Peroxide-based explosives, including TATP, HMTD, and others. Nitrate-based explosives include ammonium nitrate, urea nitrate, and others. Propellants and Taggants, including Black and Smokeless Powder, EGDN, and others. Chlorates, Perchlorates, and Sulfur-based HMEs. Heroin, Cocaine, Amphetamine, Methamphetamine, Ketamine, MDA, THC, LSD, Ecstasy, and others. Synthetic Opioids: Fentanyl, Carfentanyl, W-18, and others.	Vapor/Gas, Liquid, Solid	ng, ppm	10.0 kg	[114]

**Table 7:** Summary of color tests for the detection of explosives.

Manufacturer/ Detector Name.	Detectable Substances	The Physical State of the Sample	Limit of Detection	Weight	References
Serim Research Corporation/Discern^®^ HME Detection Kit	Explosives: Ammonium nitrate, urea nitrate, oxidizers such as chlorates, bromates, peroxides (hydrogen peroxide, HMTD, TATP), and perchlorates	Liquid, Solid	µg, ppm	data not available	[117]
Mistral Security, Inc./DropEx Plus Explosive Detection Kit	Explosives: TNT, PETN, RDX, HMX, AN, CAN, Chlorates/perchlorates	Liquid, Solid	ng	0.45 kg	[118]
Field Forensics, Inc./ELITE Test Kit Series	Explosives: AN, CAN, TNT, RDX, HMX, PETN, EGDN, all nitrate, peroxide, chlorates, and perchlorate-based explosives	Liquid, Solid	ng	2.3 kg	[119]
Mistral Security, Inc./ExPen	Explosives: ExPen 1: TNT ExPen 2: RDX, HMX, PETN ExPen 3: AN, CAN ExPen A: Chlorates ExPen P: Perchlorates, Other Chemical Targets: Nitroaromatics, nitrate esters, nitroamines, inorganic nitrates, bromates, and peroxides	Liquid, Solid	ng	0.9 kg	[120]
Haztech Systems, Inc./HazCat 2.0 Pro	Explosives: Military and common explosives, Chemical Agents: G-series, V-series, GV-series, DC, DF, QL, chlorosarin, chlorosaman, carbamates, Sulfur based (HD, T, Q), Arsenic based (L, PD, MD, ED), Nitrogen-based (HN1, HN2, HN3), essential precursors, ITF-25 TIC/TIMs, Narcotics: Opiates (morphine, codeine, heroin), brown heroin, black tar, Demerol, LSD, Amphetamines, methamphetamine, MDMA, marijuana, hashish, hash oil, cocaine, crack, PCP, Ephedrine, Other Chemical: Illicit drugs, radiological, and BWs	Liquid, Solid	ppm, mg	11.8 kg	[121]
IDenta Corp./IDenta Drug and Explosives Test Kits	Explosives: Urea nitrate, ammonium nitrate, TATP, peroxide, chlorate, bromate, perchlorate, ITF-25 TIC/TIMs: Ephedrine, acetic anhydride, MDMA precursors, GBL, Narcotics: Cocaine, heroin, marijuana, LSD, synthetic cannabinoids and cathinones, ecstasy, methamphetamine, amphetamine, ketamine, GHB, barbituates, flunitrazepam, morphine, PCP, mandrax, mephedrone, fentanyl, paracetamol, methadone	Liquid, Solid	data not available	0.040 kg	[122]
Field Forensics, Inc./Spot.On.ID™	Explosives: RDX, PETN, TNT, Ammonium nitrate. Narcotics: heroin, fentanyl, synthetic cannabinoids.	Liquid, Solid	ng	13.6 kg	[123]

**Table 8:** Operating parameters of the colorimeter SEEKERe.

Manufacturer/ Detector Name.	Detectable Substances	The Physical State of the Sample	Limit of Detection	Weight	Reference
DetectaChem/SEEKERe	Explosives, Narcotics	Liquid, Solid	ng, µg	0.197 kg	[124]

**Table 9:** Summary of the most important operational parameters of mobile FTIR/Raman detectors.

Manufacturer/Detector Name.	Detectable Substances	The Physical State of the Sample	Infrared Radiation Spectrum/Resolution/Length of Laser	Weight of the Device	References
Thermo Fisher Scientific, Inc./Gemini	Explosives, Chemical Agents (GA, GB, GD, HD, HNs, VX, RVX, others), ITF-25 TIC/TIMs, Narcotics. Other Chemical Targets	Liquid, Solid	4000–650 cm^−1^ (FTIR), 2875–250 cm^−1^ (Raman)/ 4 cm^−1^ (FTIR), 7–10.5 cm^−1^ (Raman) Laser: 785 nm	1.81 kg	[125]
908devices/VipIR	Explosives, CWA, Narcotics, Consumer Products, Chemicals	Liquid, Solid	4000–650 cm^−1^ (FTIR), 2875–250 cm^−1^ (Raman)/ 4 cm^−1^ (FTIR), 7–10.5 cm^−1^ (Raman) Laser: 785 nm	4.30 kg	[126,127]

**Table 10:** Operational parameters of IMS detectors coupled to an electrochemical sensor.

Manufacturer/ Detector Name.	Detectable Substances	The Physical State of the Sample	Limit of Detection	Weight	References
Airsense Analytics/GDA-X	Explosives (BŚT: RDX, PETN, TNT, NG, EGDN, HMTD, TATP, and others), TICs/CWAs gases	Vapor/Gas	ppt, ppb, ppm, ng	4.2 kg	[128]
Environics Oy/ChemProX	Explosives, Chemical Agents, ITF-25 TIC/TIMs, Narcotics, Other Chemical Targets	Aerosol/Gas	Data not available	0.73 kg	[129]

## Data Availability

All the data can be found in the references.

## Abbreviations

The following abbreviations are used in this manuscript:

CWA	Chemical Warfare Agents
TIC/TIMs	TIC Toxic Industrial Chemicals, TIM Toxic Industrial Materials)
TNT	2,4,6 Trinitrotoluene
RDX	Hexogen, 1,3,5-Trinitro-1,3,5-triazinane
NG	Nitroglycerin, Propane-1,2,3-triyl Trinitrate
AN	Ammonium Nitrate
PETN	Pentaerythritol Tetranitrate, 2,2-Bis[(nitrooxy)methyl]propane-1,3-diyl Dinitrate
EGDN	Ethylene Glycol Dinitrate, Ethane-1,2-diyl Dinitrate
HMX	Octogen, 1,3,5,7-Tetranitro-1,3,5,7-tetraazacyklooktan
THC	Tetrahydrocannabinol
U-47700	(3,4-Dichlorophenyl)-[1-(2-dimethylamino)cyclohexyl]methanone, a drug from the family of synthetic opioids
GB	Sarin, Propan-2-yl Methylphosphonofluoridate
VX	S-{2-[Di(propane-2-yl)amino]ethyl} O-ethyl Methylphosphonothioate
GA	Tabun, RS)-Ethyl N,N-Dimethylphosphoramidocyanidate
GD	Soman, 3,3-Dimethylbutan-2-yl Methylphosphonofluoridate
GF	Cyclosarin, Cyclohexyl Methylphosphonofluoridate
AC	Hydrogen Cyanide, Methanenitrile
CG	Phosgene, Carbonyl Dichloride
CX	Phosgene Oxime, 1,1-Dichloro-N-hydroxymethanimine
HD	Mustard Gas, 1-Chloro-2-[(2-chloroethyl)sulfanyl]ethane
HL	A Mixture of Mustard Gas with Lewisite
PD	Phenyldichloroarsine, Dichloro(fenylo)arsyna
L	Lewisite, [(E)-2-Chloroethen-1-yl]arsonous Dichloride
HN	Nitrogen Mustards, (Bis(2-chloroethyl)ethylamine)
HCN	Hydrogen Cyanide, Formonitrile
HCl	Hydrochloric Acid
NH_3_	Ammonia
CH_4_	Methane
HF	Hydrofluoric Acid
As	Arsenic
TATP	Triacetonetriperoxide, 3,3-Dimethyl-1,2-dioxacyclopropane
DMNB	2,3-Dimethyl-2,3-dinitrobutane
ANFO	Ammonium nitrate/Fuel oil
ppm	Parts Per Million
ppb	Parts Per Billion
ppt	Parts Per Trillion
MDMA	3,4-Methylenedioxymethamphetamine
MDA	3,4-Methylenedioxyamphetamine
PCP	Phencyclidine
PMA	4-Methoxyamphetamine
GHB	4-Hydroxybutyric acid
SERS	Surface-Enhanced Raman Spectroscopy
